# Knowing one's place: a free-energy approach to pattern regulation

**DOI:** 10.1098/rsif.2014.1383

**Published:** 2015-04-06

**Authors:** Karl Friston, Michael Levin, Biswa Sengupta, Giovanni Pezzulo

**Affiliations:** 1 The Wellcome Trust Centre for Neuroimaging, Institute of Neurology, Queen Square, London, UK; 2 Biology Department, Center for Regenerative and Developmental Biology, Tufts University, Medford, USA; 3 Institute of Cognitive Sciences and Technologies, National Research Council, Rome, Italy

**Keywords:** active inference, morphogenesis, self-assembly, pattern formation, free energy, random attractor

## Abstract

Understanding how organisms establish their form during embryogenesis and regeneration represents a major knowledge gap in biological pattern formation. It has been recently suggested that morphogenesis could be understood in terms of cellular information processing and the ability of cell groups to model shape. Here, we offer a proof of principle that self-assembly is an emergent property of cells that share a common (genetic and epigenetic) model of organismal form. This behaviour is formulated in terms of variational free-energy minimization—of the sort that has been used to explain action and perception in neuroscience. In brief, casting the minimization of thermodynamic free energy in terms of variational free energy allows one to interpret (the dynamics of) a system as inferring the causes of its inputs—and acting to resolve uncertainty about those causes. This novel perspective on the coordination of migration and differentiation of cells suggests an interpretation of genetic codes as parametrizing a generative model—predicting the signals sensed by cells in the target morphology—and epigenetic processes as the subsequent inversion of that model. This theoretical formulation may complement bottom-up strategies—that currently focus on molecular pathways—with (constructivist) top-down approaches that have proved themselves in neuroscience and cybernetics.

## 1. Introduction

One of the central problems of biology is the origin and control of shape [1–3]. How do cells cooperate to build highly complex three-dimensional structures during embryogenesis? How are self-limiting growth and remodelling harnessed for the regeneration of limbs, eyes and brains in animals such as salamanders [4]? Understanding how to induce specific changes in large-scale shape (not only gene expression or cell differentiation) is crucial for basic developmental and evolutionary biology—and is a fundamental requirement for radical advances in regenerative medicine and synthetic bioengineering [5–9].

Although we now know an enormous amount about the molecular components *required* for patterning [3,10–12], it is currently unclear how the macromolecular and cellular constituents of a system orchestrate themselves to generate a specific structure and function [12–16]. Importantly, numerous organisms are able to readjust their pattern to a specific target morphology despite drastic external perturbations and are able to stop growth and remodelling when the correct shape is reached [17–19]. It is largely unknown how cell behaviours are coordinated to reach correct large-scale morphologies, and how the system is able to stop when the correct structure is complete. There are very few constructivist models that show what dynamics are *sufficient* for complex patterns to arise and be remodelled until a target anatomy results. The current focus on specific protein pathways has engendered a knowledge gap: high-resolution genetic data have not, in general, being able to specify what large-scale shape—or deformations of that shape—can or will occur. An important corollary is that it is difficult to know how or which of the myriad of low-level components (genes or proteins) must be tweaked to obtain a specific patterning change [16]. This holds back progress in the biomedicine of birth defects and regeneration, which seek to induce the formation of coherent organs of correct size, shape and orientation—not merely gene activity profiles or stem-cell differentiation.

In contrast to the near-exclusive pursuit of bottom-up strategies, a complementary approach has recently been suggested [20,21]. It is possible that top-down models [22–25] could parsimoniously explain high-level phenomena [26], such as coordination of cell behaviour towards a specific topological arrangement, and could provide strategies for exploiting developmental modularity and pluripotentiality to achieve desired changes in large-scale shape. Despite the successful use of goal-seeking models in cybernetics [27], physics [28–30], and cognitive neuroscience [31,32], these principled approaches have not been applied to morphogenesis. Here, we explore a specific application of these ideas, modelling morphogenesis via an optimality principle.

In this paper, we pursue the notion that morphogenetic self-organization requires each cell to have an implicit model of its place in the final morphology [21]—and that self-assembly is the process of moving to sample local signals that are predicted by that model. In other words, we consider biologically plausible solutions to the inverse problem of how cells attain a target morphology, based upon a forward or generative model of the signals they should sense after they have attained that form [16]. In brief, we formalize the solution in terms of an extremum or optimality principle; namely, the minimization of free energy. This minimization is an inherent aspect of self-organization at many levels. For example, protein folding that minimizes thermodynamic free energy [33]. However, we consider free-energy minimization with a twist: by re-writing the minimization of thermodynamic free energy in terms of a variational free energy from information theory, one can interpret self-organization in a Bayesian sense. Effectively, minimizing variational free energy is equivalent to maximizing the (Bayesian) evidence for a model that is contained in the signals (data) sampled by a system. This enables one to talk about self-organization in terms of inference and probabilistic beliefs that are implicit in its exchange with its local environment. This perspective is based upon a long history of theoretical work in the neurosciences that attempts to formulate action and perception in terms of conscious and unconscious inference [34–41]. In recent years, the ensuing variational free-energy principle has been applied to cellular [42] and pre-biotic self-organization [43]: in which the environment supplies (sensory) signals to a system's internal states which, in turn, inform action on the environment. Both the changes in internal and active states minimize variational free energy, resulting in Bayes-optimal perception and action, respectively. This is known as *active inference*.

Here, we ask whether the same principles can explain self-assembly in the setting of morphogenesis. This is a particularly difficult problem because, unlike generic pattern formation, morphogenesis implies a pre-determined pattern or form to which an ensemble of cells should converge. However, from the point of view of any one cell, the remaining cells constitute external or environmental states that can only be inferred through intercellular signalling; molecular or electrochemical [44]. This means that each cell can only infer its place in the target morphology when all the cells have reached their target destination—and are releasing the appropriate (chemotactic) signals. This presents a difficult chicken and egg (inverse) problem that requires each cell to differentiate itself from all other cells, so that it can release signals that enable other cells to differentiate themselves.

One solution to this hard problem of self-assembly is to assume that every (undifferentiated) cell has the same model of the cellular ensemble, which it uses to predict the signals it should encounter at each location in the target form. At the beginning of morphogenesis, all the cells are thus identical: they possess the same model and implicit (stem-cell like) pluripotentiality, and know nothing about their past locations or their ultimate fate. If each cell then minimizes variational free energy then it should, in principle, come to infer its unique place in the ensemble and behave accordingly. This is guaranteed because the minimum of variational free energy is obtained when each cell is in a unique location and has correctly inferred its place. At this point, it will express the appropriate signals and fulfil the predictions of all other cells; thereby, maximizing the evidence for its model of the ensemble (and minimizing the free energy of the ensemble). This behaviour can be seen as autonomous, self-constructing or ‘autopoietic’ in the sense of Maturana & Varela [45]. In fact, active inference (in many respects) can be regarded as a formalization of autopoiesis.

In what follows, we present some simple simulations of cell migration and differentiation that provide a proof of principle that self-assembly can be understood in these terms. The resulting dynamics paint a relatively simple picture, where the parameters of each cell's model are genetically encoded—telling each cell how it should behave (what it should express) if it knew its place within the ensemble. One can then associate intracellular signalling—in response to signal receptor activation—with inferring its place or identity. This inference then leads to the transcription and release of appropriate molecular signals that induce intracellular signalling in other cells. This (signalling-dependent) transcription could be a metaphor for epigenetic processes. We first briefly review the fundaments of (thermodynamic) free-energy minimization in coupled (random dynamical) systems and how these can be cast as active (Bayesian) inference. We then use this formalism to simulate the morphogenesis of a simple organism (with a head, body and tail) to illustrate the emergent behaviour. Finally, we simulate some experimental perturbations to illustrate the predicted consequences in terms of regeneration and dysmorphogenesis.

## 2. Generalized dynamics

We will consider self-assembly in (weakly mixing ergodic) random dynamical systems described by stochastic differential equations of the following form:2.1
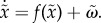


Here, the flow of generalized states 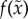 is subject to random fluctuations 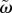. Generalized states 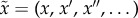 comprise the states *per se*, their motion, velocity, acceleration and so on. In essence, these equations specify probability distributions over paths in generalized coordinates of motion. We can now use the Helmholtz decomposition (a.k.a. the fundamental theorem of vector calculus) to express the flow in terms of a divergence-free component and a curl-free descent on a scalar Lagrangian 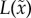 or Lyapunov function:2.2
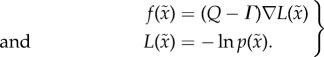
This is known as the standard form [46], where the diffusion tensor *Γ* is half the covariance (amplitude) of the random fluctuations and the matrix *Q* satisfies 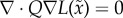. Because the system is ergodic (and weakly mixing), it will converge over time to an invariant set of states called a *pullback* or *random global attractor* [47,48]. The associated *ergodic density* 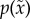 is the solution to the Fokker–Planck equation describing the evolution of the probability density over states. It is straightforward to show that 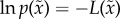 is the solution to the Fokker–Planck equation [49]. In short, any (weakly mixing ergodic) random dynamical system can be formulated as a generalized ascent on the log likelihood of its trajectories. However, this formulation does not distinguish the states of the self-organizing system from the states of its local environment. To do this, we have to consider how the states of a system are separated from its environment (e.g. heat bath). This calls on the notion of a Markov blanket.

## 3. Generalized dynamics and active inference

**Figure 1.**
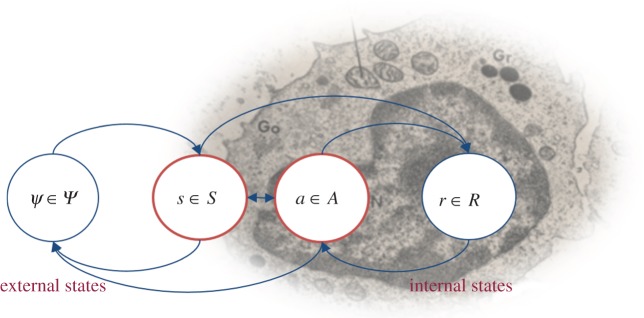
Markov blankets and active inference. This schematic illustrates the partition of states into internal states and hidden or external states that are separated by a Markov blanket—comprising sensory and active states. The internal states can be associated with the intracellular states of a cell, while sensory states become the surface states of the cell membrane overlying active states (e.g. the actin filaments of the cytoskeleton). The ensuing self-organization of internal states then corresponds to perception, while action couples internal states back to external states. See table 1 for a definition of variables. (Online version in colour.)

A Markov blanket is a set of states that separates two other sets in a statistical sense. The term was introduced in the context of Bayesian networks or graphs [50] and refers to the children of a set (the set of states that are influenced), its parents (the set of states that influence it) and the other parents of its children. A Markov blanket induces a partition of states into *internal states* and *external* states that are hidden (insulated) from the internal (insular) states by the Markov blanket. For example, the surface of a cell may constitute a Markov blanket separating intracellular (internal) and extracellular (external) states [43,51]. Statistically speaking, external states can only be seen vicariously by the internal states, through the Markov blanket. The Markov blanket itself can be partitioned into two sets that are, and are not, children of external states. We will refer to these as surface or *sensory states* and actuator or *active states*, respectively. Put simply, the existence of a Markov blanket *S* × *A* implies a partition of states into external, sensory, active and internal states: 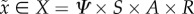. External states cause sensory states that influence—but are not influenced by—internal states, whereas internal states cause active states that influence—but are not influenced by—external states. Crucially, the dependencies induced by Markov blankets create a circular causality that is reminiscent of the action–perception cycle (figure 1).

**Table 1.** Definitions of the tuple 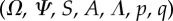 underlying active inference.

*a sample space* *Ω* or non-empty set from which random fluctuations or outcomes 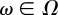 are drawn
*external states* 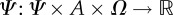 —hidden states of the world that cause sensory states and depend on action
*sensory states* 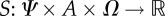 —signals that constitute a probabilistic mapping from action and external states
*active states* 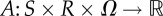 —action that depends on sensory and internal states
*internal states* 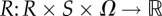 —representational states that cause action and depend on sensory states
*ergodic density* 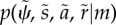 —a probability density function over external 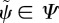 , sensory 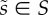 , active 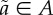 and internal states 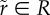 for a Markov blanket denoted by *m*
*variational density* 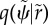 —an arbitrary probability density function over external states that is parametrized by internal states

We can now consider the dependencies among states implied by the Markov blanket, in terms of their equations of motion. In particular, we are interested in the flow of internal and active states that constitutes the systems response to sensory signals:3.1
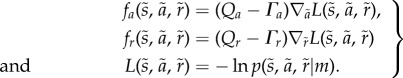


See [43] for details. This equation is the homologue of equation (2.2) for internal and active states. It says that their flow performs a generalized gradient ascent on the *marginal* ergodic density over internal states and their Markov blanket denoted by *m*. This means we can describe the (open) system in terms of a Lagrangian of the system's states that we will associate with its *thermodynamic free energy*—in the setting of the stochastic thermodynamics of non-equilibrium steady states [52].

On this point, one could stop and simply marvel at evolution for having selected equations of motion with attractors that have the intricate forms seen in biotic systems (e.g. protein folding, morphogenesis and pattern formation). These attracting forms are described probabilistically in terms of the thermodynamic free energy, describing the probability over the systems internal states and Markov blanket. Although we know this Lagrangian exists, it is practically (almost) impossible to evaluate its form. However, there is an alternative formulation of equation (3.1) that allows one to describe the flow in terms of a probabilistic model of how a system thinks it should behave. This formulation is based on the following lemma:

**Lemma 3.1**

(*free energy*): *for any random dynamical system with a Markov blanket and Lagrangian* 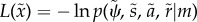, *there is a variational free energy* 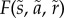 *that describes the flow of internal and active states as a generalized descent*3.2
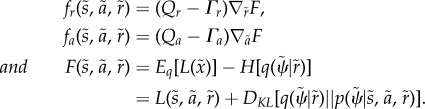

**Proof.**

See [43]. This lemma says that if one interprets internal states as parametrizing some arbitrary (variational) density or Bayesian beliefs 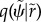 about external states, then the dynamics of internal and active states can be described as a gradient descent on variational free energy. Importantly, this free energy is a function of states that constitute the system; namely, the internal states and their Markov blanket. Variational free energy was introduced by Feynman to solve difficult integration problems in path integral formulations of quantum physics [53]. This is also the free energy bound on log *model evidence* that is used extensively in *approximate Bayesian inference*; for example, variational Bayes and ensemble learning [54–56].

The expressions for variational free energy above highlight its Bayesian interpretation: the first equality expresses free energy as the expected Lagrangian (Gibbs energy) minus the entropy of the variational density. The second equality shows that variational free energy is the thermodynamic free energy plus a *relative entropy* or Kullback–Leibler divergence [57] between the variational density and the posterior density over external states. The solution to equation (3.2) implies the internal states minimize free energy rendering the divergence zero (by Gibbs inequality). This means the variational free energy becomes the thermodynamic free energy—and the variational density becomes the posterior density. In this setting, the thermodynamic free energy is also known as the log *marginal likelihood* or log model evidence. In short, the internal states will appear to engage in Bayesian inference, effectively inferring the (external) causes of sensory states. Furthermore, the active states are complicit in this inference, sampling sensory states that maximize model evidence: in other words, selecting sensations that the system expects. This is *active inference*, in which internal states and action minimize free energy—or maximize model evidence—in a way that is consistent with the good regulator theorem and related treatments of self-organization [49,58–61].

The variational formulation above speaks directly to two fundamental observations made at the inception of cybernetics; namely, every good regulator is a model of its environment, and the law of requisite variety [58,62]. The first observation is endorsed by the fact that variational free energy is a functional of a probabilistic model of how sensory states are generated by external states. The law of requisite variety follows from the fact that the variational density must be encoded by internal states whose cardinality equals or exceeds that of the sufficient statistics of the posterior density. This is necessary to eliminate the divergence between the variational and posterior densities. See [63] for a closely related discussion of information-based optimality criteria for control systems. The perspective afforded by the good regulator theorem highlights the fact that the (open) system can become the author of its environment. In other words, it can control external or hidden states through action—such that they fulfil the predictions of the generative model. Indeed, as we will see later, the hidden states of the generative model do not even need to exist—provided their sensory consequences can be mediated through action. This is important because it allows one to specify the thermodynamic free energy in terms of a generative model, thereby specifying sets of attracting states (e.g. target morphologies) that can have quite complicated forms.

Equipped with this (active inference) formulation, we can now simulate self-assembly by integrating equation (3.2) given a (probabilistic generative) model that entails beliefs about its environment. In other words, we only need to specify the generative model 
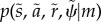
 and the dynamics of the environment 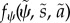 and 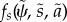 to completely specify the requisite equations of motion for the system and its environment. Generally, the model is specified in terms of nonlinear mappings with additive noise:3.3
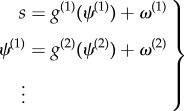


Gaussian assumptions (parametrized by their precision or inverse variance) about the random fluctuations 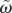 prescribe the *likelihood* and *priors* over external states that define the Lagrangian or generative model3.4
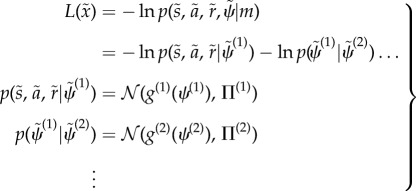
where 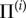 correspond to the precision or inverse variance of the random fluctuations. In what follows, we integrated equation (3.2) using the Matlab routine **spm_ADEM.m** in the **SPM** academic freeware.^1^ This scheme uses the Laplace assumption 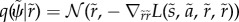 and associates divergence-free flow within generalized motion 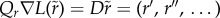. The resulting scheme can be regarded as a generalized Bayesian filter, in which the internal states become the expected values of the external or hidden states, see [64] for details. This scheme has been used in several papers to simulate active inference in the neurosciences. In what follows, we use it to simulate self-assembly—and how this illuminates the role of genetics and epigenetics in morphogenesis. These simulations are offered as a proof of principle that the above scheme provides a sufficient explanation for (simple) self-assembly.

## 4. Simulating self-assembly

**Figure 2.**
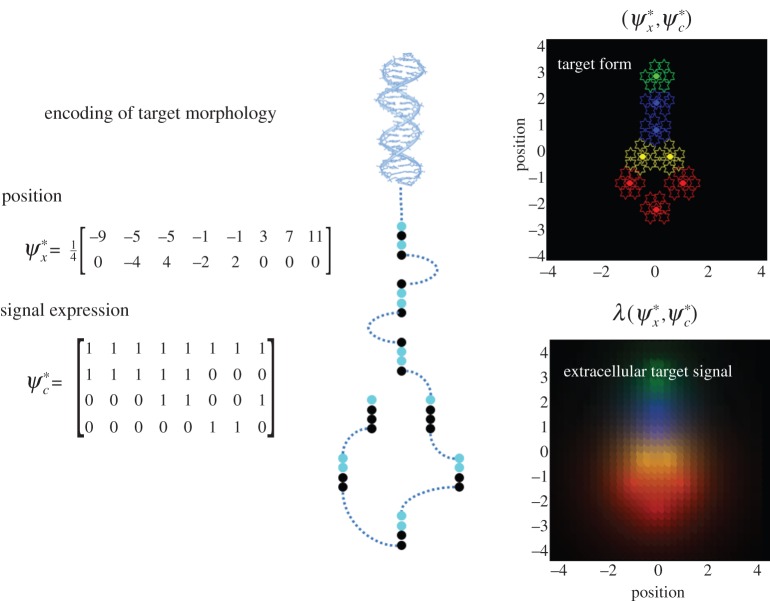
Target morphology. The right panels show the encoding of target locations (upper left) and cell type (lower left). This encoding can be regarded as a genetic code specifying the chemotactic signals expressed by each cell, which is associated with a specific—possibly place coded—location. In other words, the intracellular location of the code may encode the extracellular location, as indicated in the schematic (middle panel) associating signal expression with binary codons. The corresponding configuration is shown in the upper right panel, where the last three signal expressions are used to differentiate cells (using a red–green–blue colour scheme). The locations of the eight clones are shown with filled stars. The lower right panel shows the target concentrations that would be sensed at each location under this target configuration. (Online version in colour.)

We simulated morphogenesis given a target morphology specified in terms of the location and differentiation of eight clones or cells shown in figure 2 (a simple form with a head, body and tail). Here, we focus on the migration and differentiation of each clone prior to subsequent proliferation (filled cells in the upper right panel of figure 2). We deliberately stripped down the problem to its bare essentials to illustrate the nature of the dynamics, which will be used in the final section to make predictions about the results of various interventions. In brief, starting from an ensemble of undifferentiated cells at the same location, we wanted to simulate their migration and differentiation using minimization of free energy. For simplicity, we did not consider cell division; however, the ensuing behaviour showed distinct phases of migration and differentiation that was mediated exclusively by extracellular signals: e.g. chemotactic signals or slow electrochemical coupling [44]. Crucially, at the beginning of morphogenesis all the cells were identical: although they all possessed the same model and implicit (stem-cell like) pluripotentiality, they knew nothing about where they were or their ultimate fate.

A key aspect of the resulting self-assembly is a circular causality that follows from modelling multiple cells, where each cell is constituted by internal (intracellular) states and their Markov blanket. The sensory states of the Markov blanket were limited to chemoreceptor samples of extrinsic (extracellular) and intrinsic (intracellular) concentrations, while the active states could either cause cell migration (on a two-dimensional surface) or the release of chemotactic signals. This is an interesting set-up because the external states of one cell are the active states of other cells. This means we are effectively simulating a multi-system or multi-agent problem that, as we will see, necessarily entails some form of communication or generalized synchrony among cells.

By framing self-assembly as active inference, we can now functionally talk about a cell's beliefs and actions in the following way: each cell possesses a generative model that encodes (genetic) beliefs about the chemotactic signals it should sense and express if it occupied a particular place in the target form. However, it has to first infer its identity on the basis of chemotactic signals—enabling it to predict the signals it should sense and express. Action can then fulfil these predictions by moving over concentration gradients to match extracellular predictions—and releasing signals to match intracellular predictions. Clearly, this would be a fairly robust (and trivial) scheme if all the other cells were in their target locations and were expressing the appropriate signals; however, at the beginning of morphogenesis they are not. This is where the circular causality comes in. In order to reach the target form, each cell has to move to an appropriate location based upon chemotactic concentration gradients. However, these gradients depend upon cell migration. In other words, self-assembly requires each cell to ‘know its place’ so that the population can establish a chemotactic frame of reference that enables each cell to ‘know its place’. This is the problem solved through minimizing free energy, where free energy is minimized when, and only when, every cell has associated itself with a unique target location. Note that, after differentiation, every cell has to infer a unique identity without access to the beliefs of other cells. This hard problem is finessed by the embodied nature of active inference: because a cell can only be in one place at a time there is a unique free-energy minimum, where every cell knows its respective place. While this may sound abstract, it is relatively straightforward to simulate self-assembly and cast morphogenesis in terms of familiar processes such as genetic pathways, epigenetic influences and biophysical or chemical guidance cues.

In detail, we simulated very simple environmental dynamics with external states that comprised the location of each cell 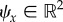 and its release of four chemotactic signals 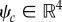, which were the corresponding active states of each cell. More realistic simulations would model cell migration and signalling as a function of action; however, we will assume action is sufficiently fast to use the adiabatic approximation 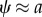: i.e. the solution to 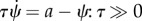. Sensory states corresponded to chemotactic concentrations of intracellular, exogenous and extracellular signals. Here we assume the existence of exogenous (linear) concentration gradients, which we will relax later4.1
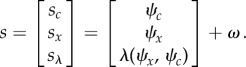


The signal receptor noise was set at very low levels with a log precision of −16. The function 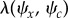 returns extracellular signal levels generated by all cells assuming a monoexponential spatial decay of concentration for each of the signals: where, for the *i*th cell4.2
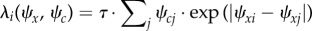
where, 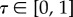 is developmental time and models a linear increase in sensitivity to extracellular signals (e.g. the progressive expression of cell surface receptors over time). Finally, each cell is assumed to have the same generative model specified in terms of the mapping from hidden states to sensations4.3
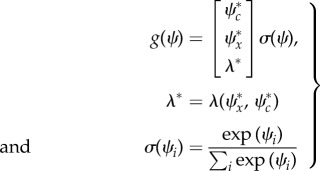
where, the matrices 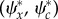 correspond to target locations and the combinations of the four signals expressed at these locations, respectively. The (softmax) function of hidden states *σ*(*ψ*) returns expectations about the identity of each cell. These expectations enable the cell to predict which chemotactic signals it should express and sense. We assumed (zero mean) Gaussian priors over the hidden states with a small precision 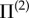 (with a log precision of minus two). This means that the cells have prior beliefs that they have a high expectation of being a particular clone but they do not know which clone they only belong to. This form of prior is ubiquitous in generative models of sparse causes (e.g. [65]). The resulting model is extremely simple and has no hierarchical structure, enabling us to drop the superscripts of equation (3.2).

This generative model or Lagrangian produces remarkably simple dynamics for internal and external states (suppressing higher order terms and using 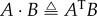):4.4
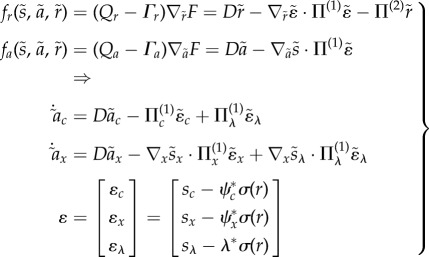
where, 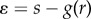 is the prediction error at the level of chemotactic signal receptors whose gradients are 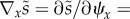 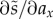. The signal precision 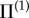 had a log precision of two, where the precisions over (generalized) motion modelled random fluctuations with a Gaussian autocorrelation function of one. These equations have some straightforward interpretations: the internal states organize themselves to minimize (precision weighted) prediction error based upon predictions. These predictions are weighted mixtures of target locations and concentrations encoded by 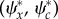. If we associate this encoding with a genetic code, then its transcription into predictions can be associated with epigenetic processes (i.e. intracellular signalling-dependent transcription). Figure 2 illustrates this graphically using the target morphology assumed for the simulations. Here, different segments of the target form are associated with four cell types, defined in terms of the combination of signals expressed.

The updates for action are even simpler. Active states control the expression of chemotactic signals and cell migration. Signal expression simply attempts to close the gap between the predicted and detected signals, while migration is driven by local concentration gradients sensed by the cell. It is interesting to note that these gradients require a distribution of receptors over a spatially extensive surface or sensory epithelia, which is characteristic of living organisms. Furthermore, it requires the spatial scale of cells to be non-trivial in relation to chemotactic gradients; although see [66] for a discussion of active sampling in this context. Heuristically speaking, the cell moves to fulfil its expectations about the signals it thinks it should encounter, while expressing the signals associated with its current beliefs about its place in the target ensemble.

**Figure 3.**
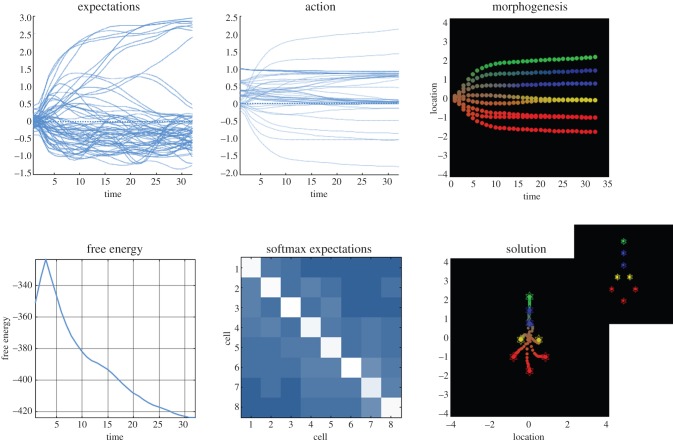
Self-assembly. This figure shows the results of a simulation in terms of the solution or trajectory implied by active inference. This simulation used a local linear approximation to integrate the generalized descent on free energy (equation (4.3)) in 32 time steps, using the target morphology for eight cells in figure 2. Each time step can be thought of as modelling migration and differentiation over several minutes. The upper panels show the time courses of expectations about cell identity (left), the associated active states mediating migration and signal expression (middle) and the resulting trajectories; projected onto the first (vertical) direction—and colour-coded to show differentiation. These trajectories progressively minimize free energy (lower left panel), resulting in expectations that establish a relatively unique differentiation of the ensemble (lower middle panel). This panel shows the softmax function of expectations for each of eight cells, which can be interpreted as the posterior beliefs that each cell (column) occupies a particular place in the ensemble (rows). The columns have been reordered so that the maximum in each row lies along the leading diagonal. The lower right panel shows the ensuing configuration using the same format as figure 2. Here, the trajectory is shown in small circles (for each time step). The insert corresponds to the target configuration. (Online version in colour.)

Figure 3 shows the resulting self-assembly using the target morphology shown in figure 2. These simulations used 32 time steps (each corresponding to several minutes). The resulting trajectories show several interesting features. First, there is a rapid dispersion and migration of the cells to their target locations, followed by a differentiation into the respective cell types (at about the eighth time step). This migration and differentiation is accompanied by a profound reduction in free energy—as the solution converges towards the target configuration. Note the initial increase in free energy as cells disperse a bit too quickly on the first iteration (as often seen with nonlinear generative models). The free energy here is the free energy of the ensemble or the sum of the free energy of each cell (because the variational and posterior densities are conditionally independent given sensory signals). All the cells started at the same location and with undifferentiated expectations about their fate (using small random expectations with a log precision of minus four). In this example, the self-assembly is not perfect but reproduces the overall form and differentiation into a head, body and tail cell types. We terminated the simulation prematurely to illustrate the partial resolution of uncertainty about cellular identity implicit in the expectations encoded by the internal states. These are shown in the lower middle panel of figure 3 for all cells (after application of the softmax function) and can be interpreted as a confusion matrix. The off-diagonal block structure of this confusion matrix shows that, as one might expect, there is a mild confusion between head and body cells, and between body and tail cells (but not between head and tail cells). This confusion resolves after continued differentiation (results not shown).

These results are compelling in the sense that they show cells can resolve ambiguity about their fate, even when that resolution depends upon the context established by other (equally ambiguous) cells. Subsequent work will increase these models’ biological realism by associating various quantities with intracellular signalling and transcription, to enable testing of specific predictions about the outcomes of various experimental interventions. To illustrate the potential of this sort of modelling, we conclude with a brief simulation of regeneration and dysmorphogenesis to illustrate the sorts of behaviour the simple system above can exhibit.

## 5. Simulating regeneration and dysmorphogenesis

Real biological tissues demonstrate remarkable self-repair and dynamic reconfiguration. For example, planarian regeneration provides a fascinating model of reconfiguration following removal of body parts or complete bisection (reviewed in [4]); early embryos of many species are likewise regulative and fully remodel following bisection. To illustrate this sort of behaviour, we simulated morphogenesis for eight time steps and then cut the partially differentiated embryo into two. We then simulated cell division by replicating the cells in each half (retaining their locations and partial differentiation) and continued integrating the morphogenetic scheme for each part separately.

**Figure 4.**
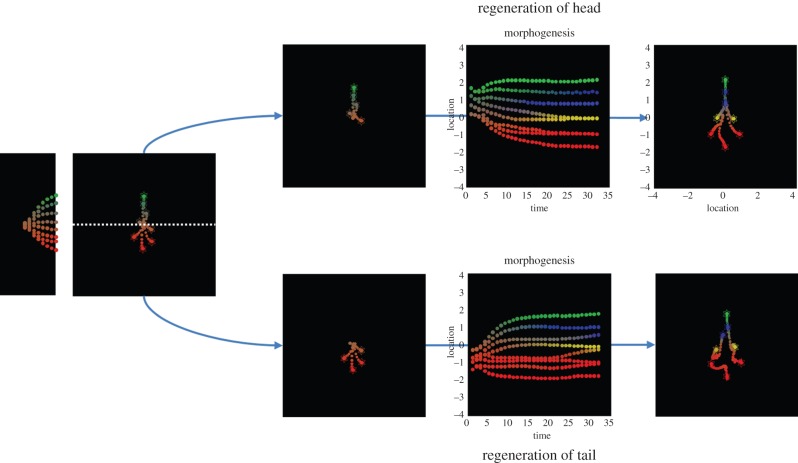
Simulating regeneration. This figure reports simulated interventions that induce dynamic reorganization. The left panels show the normal self-assembly of eight cells or clones over iterations using the format of the previous figures (with a veridical generative model). The right panels show the development of the ensemble after it has been split into two (by the dashed line) and duplicated. The upper panels show the fate of the cells that were destined to be the tail, whereas the lower panels show the corresponding development of cells destined to be the head. Both of these (split embryo) clones dynamically reconfigure themselves to produce the target morphology, although the head cells take slightly longer before it ultimately converges (results not shown). (Online version in colour.)

The left panels of figure 4 shows normal morphogenesis over eight time steps, while the upper row shows the subsequent development of the progenitor tail cells over 32 iterations. This effectively simulates the regeneration of a head. The complementary development of the head cells can be seen in the lower panels. One can see that the cells (that were originally destined to become tail and head cells) undergo a slight dedifferentiation before recovering to produce the target morphology. In this example, the head of the split embryo takes slightly longer to attain the final form. This example illustrates the pluripotential nature of the cells and the ability of self-assembly to recover from fairly drastic interventions.

**Figure 5.**
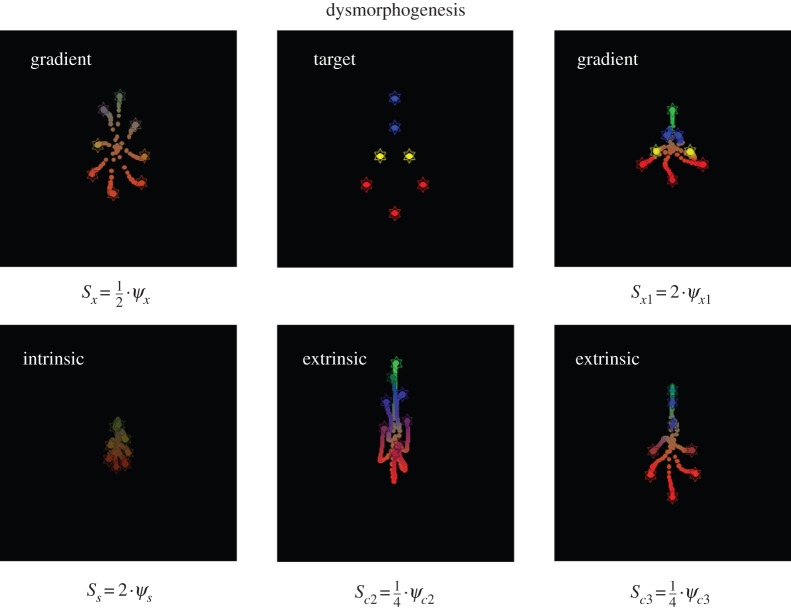
Perturbing self-assembly. The upper right and left panels show deviations from the target configuration when the self-assembly is confounded by changing the concentration of (or sensitivity to) exogenous gradients. The first intervention illustrates a failure of migration and differentiation that results from non-specific suppression of exogenous signals, while selectively increasing the vertical gradient produces compression along the corresponding direction. More exotic forms of dysmorphogenesis result when decreasing the intracellular (intrinsic: lower left panel) and extracellular (extrinsic: lower right panels) receptor sensitivities. (Online version in colour.)

To illustrate dysmorphogenesis (e.g. induced birth defects), we repeated the above simulations while changing the influence of extracellular signals—without changing the generative model (genetic and epigenetic processes). Figure 5 shows a variety of abnormal forms when changing the levels of (or sensitivity to) exogenous, intracellular and extracellular signals. For example, when the sensitivity to exogenous gradients is suppressed, the cells think they have not migrated sufficiently to differentiate and remain confused about their identity. Conversely, if we increase the sensitivity to the vertical gradient, the cells migrate over smaller vertical distances resulting in a vertical compression of the final form. Doubling the sensitivity to intracellular signals causes a failure of migration and differentiation and generalized atrophy. More selective interventions produce a dysmorphogenesis of various segments of the target morphology: reducing sensitivity to the second chemotactic signal—that is expressed by head cells—reduces the size of the head. Similarly, reducing sensitivity to the third signal induces a selective failure of body cells to differentiate. These simulations are not meant to be exhaustive but illustrate the predictions one could make following experimental manipulations (e.g. pharmacological) of intercellular signalling.

## 6. Conclusion

In summary, we have introduced a variational (free energy) formulation of ergodic systems and have established a proof of principle (through simulations) that this formulation can explain a simple form of self-assembly towards a specific pattern. We have framed this in terms of morphogenesis, relating the generative model implicit in any self-organizing system to genetic and epigenetic processes. There are clearly many areas for subsequent expansion of this work; for example, cell division and neoplasia: see [67]—and the vast knowledge accumulated about the molecular biology and biophysics of morphogenesis. Furthermore, we have only considered generative models that have a fixed point attractor. In future work, it would be interesting to include equations of motion in the generative model, such that certain cell types could express fast dynamics and generate dynamic behaviours (like pulsation) following differentiation. Finally, future work should explore whether and how the self-organization and autopoietic dynamics that we demonstrated in the morphogenetic domain generalize to larger-scale phenomena such as brains and societies, as has been variously proposed [45,68].

The premise underlying active inference is based upon a variational free energy that is distinct from thermodynamic free energy in physics. In fact, strictly speaking, the formalism used in this paper ‘sits on top of’ classical physics. This is because we only make one assumption; namely, that the world can be described as a random dynamical system. Everything follows from this, using standard results from probability theory. So how does variational free energy relate to thermodynamic free energy? If we adopt the models assumed by statistical thermodynamics (e.g. canonical ensembles of microstates), then variational free-energy minimization should (and does) explain classical mechanics (e.g. [69,70]). In a similar vein, one might hope that there is a close connection with generalizations of thermodynamic free energy to far-from-equilibrium systems [71] and non-equilibrium steady state (e.g. [52,72]). This might be important in connecting variational and thermodynamic descriptions. For example, can one cast external states as a thermal reservoir and internal states as a driven system? In driven systems—with a continuous absorption of work followed by dissipation—the reliability or probability of such exchange processes might be measured by the variational free-energy, so that stable structures are formed at its minima [73]. However, questions of this sort remain an outstanding challenge.

Our simulations rest on several simplifying assumptions, such as what is coded in the cells, the (place-coded) nature of the target location, and the specifics of cell–cell communication. It is important to note that the framework does not depend explicitly on these assumptions—and extending the model to include more detailed and realistic descriptions of genetic coding and signalling is an open challenge. A potentially exciting application of these schemes is their use as observation models of real data. This would enable the model parameters to be estimated, and indeed the form of the model to be identified using model comparison. There is an established procedure for fitting models of time-series data—of the sort considered in this paper—that has been developed for the analysis of neuronal networks. This is called *dynamic causal modelling* [74,75] and uses exactly the same free-energy minimization described above. Although the nature of neuronal network and morphogenetic models may appear different, formally they are very similar: neuronal connectivity translates into kinetic rate constants (e.g. as controlled by the precisions above) and neuronal activity translates to the concentration or expression of various intracellular and extracellular signals. In principle, the application of dynamic causal modelling to empirical measurements of morphogenesis should provide a way to test specific hypotheses through Bayesian model comparison [76] and ground the above sort of modelling in a quantitative and empirical manner. It should also be pointed out that is now clear that even non-neuronal cells possess many of the same ion channel- and electrical synapse-based mechanisms as do neurons and use them for pattern formation and repair [77–82]. This suggests the possibility that significant commonalities may exist between the dynamics of neural networks and of bioelectric signalling networks that control shape determination [20,21,83].

The example in this paper uses a fairly arbitrary generative model. For example, the choice of four signals was largely aesthetic—to draw a parallel with DNA codons: the first signal is expressed by all cells and is primarily concerned with the intercellular spacing. The remaining three signals provide for eight combinations or differentiated cell types (although we have only illustrated four). One might also ask about the motivation for using an exogenous gradient. This is not a necessary component of the scheme; however, our simulations suggest that it underwrites a more robust solution for larger ensembles (with more than four clones). Furthermore, it ensures that the orientation of the target morphology is specified in relation to an extrinsic frame of reference. Having said this, introducing a hierarchical aspect to the generative model may be a more graceful way to model real morphogenesis at different spatial scales.

We have hypothesized that the parameters of each cell's model are genetically encoded. Another intriguing possibility is that the cells learn some model parameters during epigenesis and growth through selective expression of a fixed code. In other words, during growth, each cell might acquire (or adjust) the parameters of its generative model such that the target morphology emerges during epigenesis. The organism as a whole may therefore exhibit a self-modelling process—where they are essentially modelling their own growth process. A useful analogy here is self-modelling robots, which learn models of their own structure (e.g. their body morphology) and use those models to predict the sensory consequences of movement—and maintain their integrity or recover from injuries [84]. In computational neuroscience and machine learning, various methods have been proposed for this form of (structure) learning [85]. Technically, the difference between learning and inference pertains to the optimization of parameters and expected states with respect to model evidence (i.e. free energy). We have focused on inferring (hidden) states in this paper, as opposed to parameters (like log precisions). It may be worth concluding that although aspects of the target morphology are genetically encoded (in the parameters of the generative models); epigenetic processes (active inference) are indispensable for patterning. Indeed, it is possible to permanently alter the regenerative target morphology in some species; for example, perpetually two-headed planaria can be created by a brief modification of the electric synapses [44,86].

Finally, we have not considered the modelling of different phases of development or hierarchical extensions of the basic scheme. Having said this, the variational formulation offered here provides an interesting perspective on morphogenesis that allows one to talk about the beliefs and behaviour of cells that have to (collectively) solve the most difficult of inference problems as they navigate in autopoietic frame of reference. It is likely that this is just a first step on an important roadmap to formalize the notion of belief and information-processing in cells towards the efficient, top-down control of pattern formation for regenerative medicine and synthetic bioengineering applications. Perhaps the last word on this spatial inverse problem should go to Helmholtz [35], p. 384

Each movement we make by which we alter the appearance of objects should be thought of as an experiment designed to test whether we have understood correctly the invariant relations of the phenomena before us, that is, their existence in definite spatial relations.

## Endnote

1 http://www.fil.ion.ucl.ac.uk/spm/. The simulations in this paper can be reproduced by downloading the **SPM** freeware and typing DEM to access the **DEM Toolbox** graphical user interface. The requisite routines can be examined and invoked with the **morphogenesis** button. Annotated Matlab code provides details for performing simulations with different target morphologies—or different chemotactic signalling.
